# Over ten-year follow-up results of a prospective and consecutive series of primary total hip arthroplasty with an original cementless total hip prosthesis

**DOI:** 10.1007/s00264-023-06071-7

**Published:** 2023-12-28

**Authors:** Claude Schwartz, Christophe Bussiere, François Chalencon, Franck Cladiere, Philippe Forgeois, Christophe Fornasieri

**Affiliations:** 1 6 Rue du Riesling, 68230 Zimmerbach, France; 2 Centre Orthopédique de Dracy-Le-Fort, 2 Rue du Pressoir, 71640 Dracy-Le-Fort, France; 3 Centre Ortheo, 21 Boulevard Karl Marx, 42100 Saint-Etienne, France; 4 Clinique Pasteur, 180 Rue Pierre CurieGuilherand, 07500 Granges, France; 5 Clinique Saint-Amé, Rue Georges Clémenceau, 59552 Lambres Lez Douai, France; 6 Clinique Générale d’Annecy, 4 Chemin de La Tour La Reine, 74000 Annecy, France

**Keywords:** Total hip replacement, Cementless hip arthroplasty, Long-term follow-up, Anatomic form

## Abstract

**Purpose:**

High survival rates up to ten  years have been reported for non-cemented hip replacements. Publications beyond ten years have more diverse conclusions. To study the long-term survival of uncemented total hip replacement (THR), we examined a series of 125 THR, all with a minimum follow-up of ten  years.

**Methods:**

This is a prospective study of 203 patients operated for coxarthrosis between 2007 and 2011, by six senior surgeons. The original ellipsoidal stem and the impacted acetabulum were systematically cementless; the acetabulum had either a fixed ceramic or polyethylene insert, or a dual-mobility insert. At the date of the follow-up check, 44 patients were deceased and 34 patients were lost to follow-up. This left 125 complete files for our study.

**Results:**

They were a revision of the cup in four cases and a revision of the femoral stem in three cases (3.4%). The Kaplan–Meier cumulative survival rate of the THR, by considering revision for any reason as endpoint, at ten  years (120 months) is estimated at 96.6% (CI 92.7–98.7). Radiologically, on 86 analyses (68.8%) at ten  years and more reported, no significant evolution of the appearance of the cancellous bone around the acetabular cup was noted, nor any ossification. Some periprosthetic osteogenesis reactions were noted around the 1/3 distal but no periprosthetic edging.

**Conclusion:**

In this minimum ten-year follow-up study, a cementless THR with a straight ellipsoidal cementless stem and a press-fit cup provides excellent implant survival and high patient satisfaction. (Clinically felt minimal difference.)

## Introduction

High survival rates up to ten years have been reported for non-cemented hip replacements, more for the stems than for cotyloid implants [1–4]. Publications beyond ten years have more diverse conclusions about the survival of these implants [5–20]. To study the long-term survival of uncemented total hip replacement (THR), we examined a series of 203 THR, including 125 cases with a minimum ten-year follow-up (average 12 years; range 10–14 years).

## Material and methods

This is a prospective study of 203 patients operated for primary or secondary coxarthrosis between 2007 and 2011, by six senior surgeons used to this THR surgery.

Patients had a mean age of 65.4 years (26 to 86), SD 11.5. This was 57.1% women and 42.9% men. The operated side was 43.8% on the left and 56.2% on the right side. The average weight of the operated patients was 75.6 kg with an SD of 16.7 (min 41, max 150). The average BMI was 27.2 (17 to 48) with an SD of 4.8. There were 4.5% of patients with a medical history; three who had neurodegenerative diseases had a dual mobility (DM) cup implanted, without complications.

The femoral stem and the impacted acetabulum were systematically placed without cement; the acetabulum had either a fixed ceramic or polyethylene insert, or a double mobility insert.

One hundred and fifty-two (74.9%) underwent a radio-clinical control at five years. Forty-four of them (21.7%) had died within ten years of the operation and 34 patients (16.7%) were lost to follow-up during this period; that left 125 complete files for our study.

The prosthetic implants are the “Hip and Go” brand implants (FH Ortho, Heimsbrunn, France).

### Femoral stem

The femoral stem is a straight Ti6Al4V alloy rod covered by a projection of porous titanium T40, then by a layer of 100 μ hydroxyapatite.

For primary stability allowing immediate full support, we chose an elastic metaphyseal enclave in the cancellous bone.

To avoid if possible cortical contact of the stem, a source of potential pain, we preferred a flared rod of elliptical section, with antero-posterior diameter half of the frontal diameter.

**Fig. 1:**
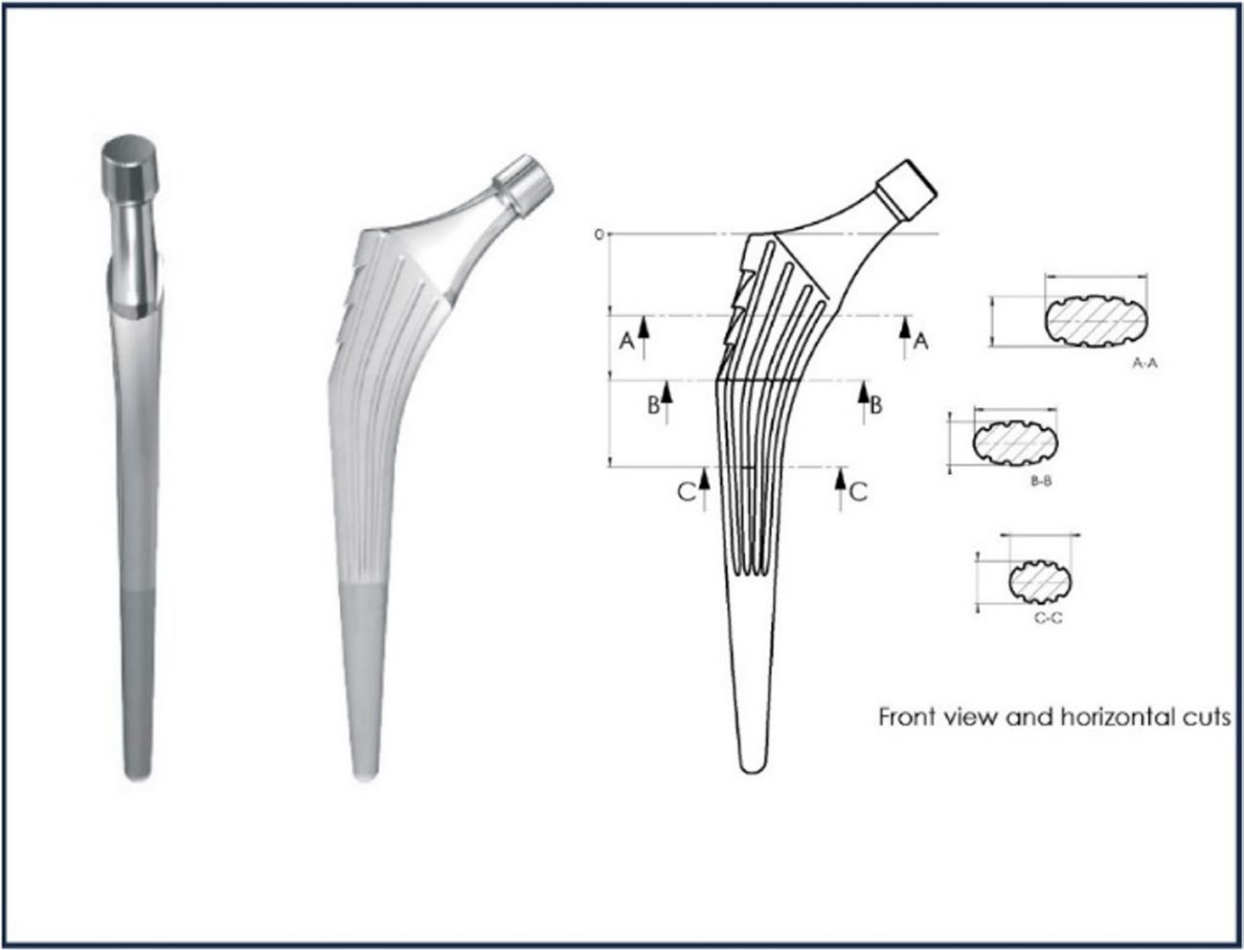
The stem has four front and rear grooves on 2/3 length and three ledges toward the trochanter

The primary press-fit fixation is improved by four front and rear grooves on 2/3 length and three ledges toward the trochanter (Fig. 1). They increase the surface area and improve secondary fixation. The stem is available in ten increasing and homothetic sizes based on 813 calques made on X-rays of coxarthritis.

**Fig. 2:**
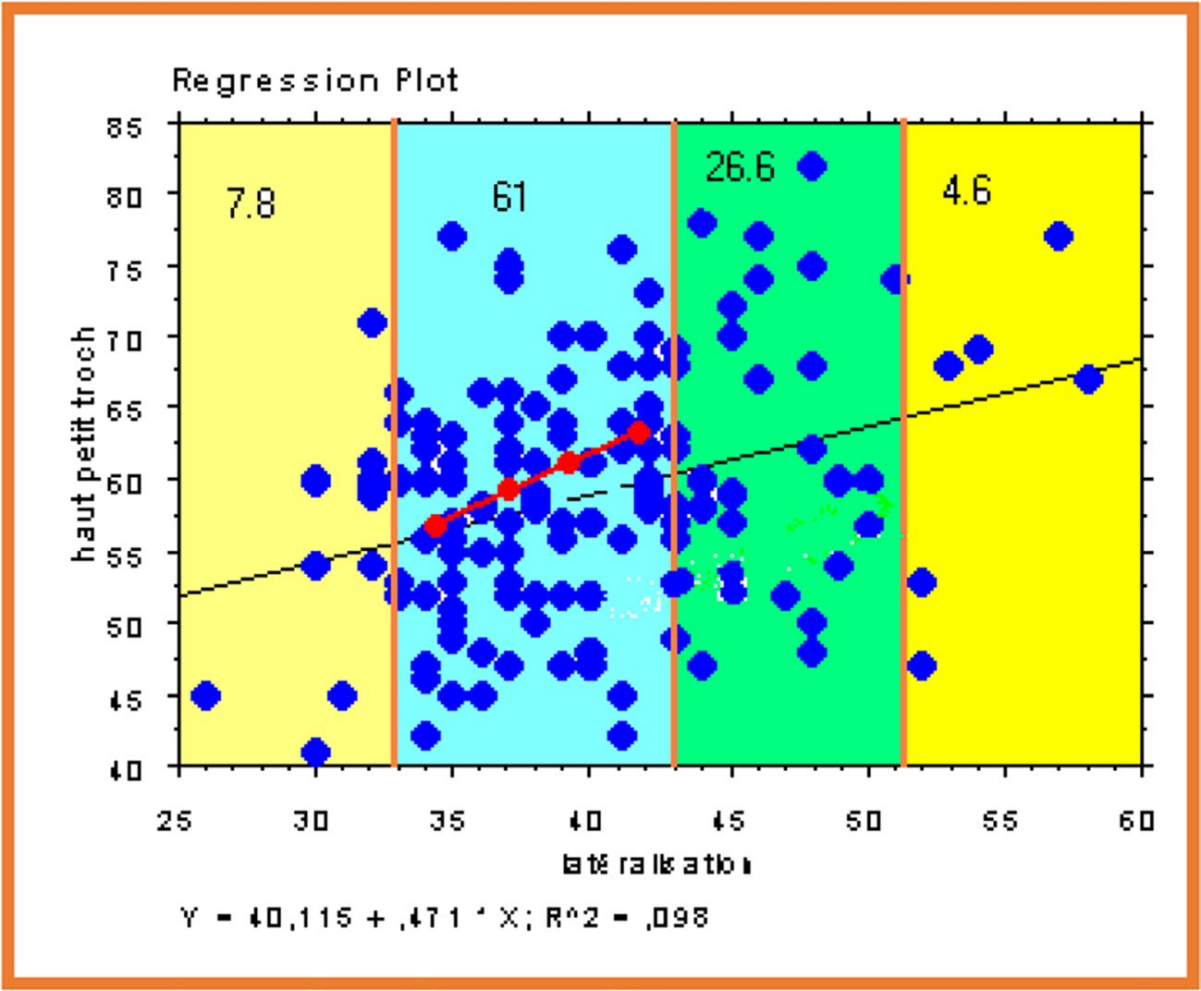
The study of the 813 X-rays determined a scatterplot of femoral head centres which allowed us to clarify their position and so the angulation and length of the prosthetic necks

**Fig. 3:**
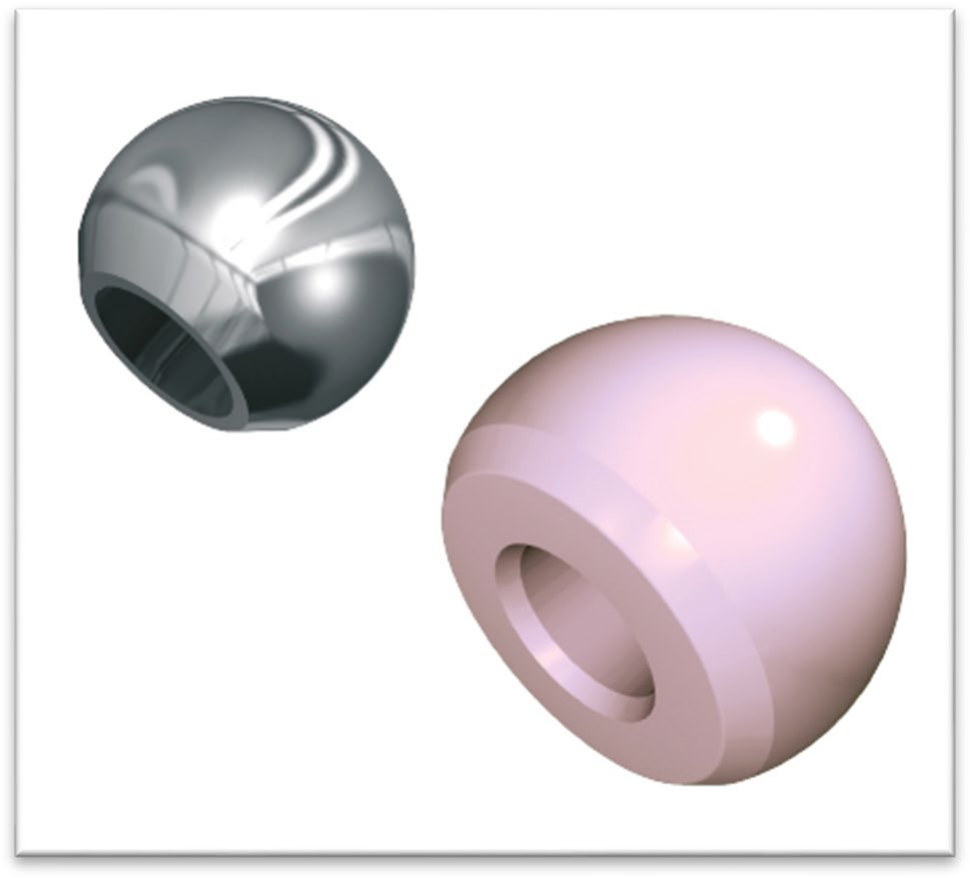
Different femoral heads with different diameters and collar lenghts

This study of the point clouds of the layers (Fig. 2) allowed us to choose the different orientations (122° and 130°) and neck lengths (always 12/14) necessary to have correct offsets, associated with a variety of head different diameter (22.2 to 36 mm), and with variable prosthetic collar insertion lengths (short, medium, long, extra-long) (Fig. 3).This neck is mirror polished, flattened antero-posterior, and refined to 11 mm below the cone.

The 10ten rasps are compaction rasps, which do not remove any bone but compress it to the periphery. They are increasingly larger, homothetic, and smaller, by a few tenths of a millimeter, than the corresponding implant.

### Acetabular cup

The hemispherical acetabulum exists in ten homothetic sizes from 48 to 62. The primary fixation in press-fit is improved by three circumferential grooves and four anti-rotation dewclaws, all in the equatorial zone. Preparation cutters are a few tenths of a millimeter smaller.

This cup exists in two varieties:

1. Made of titanium alloy for fixed inserts, either in delta ceramic or in ultra-high molecular weight polyethylene (UHMWPE) sterilized with ethylene oxide, for the rigid cup (Fig. 4).

**Fig. 4:**
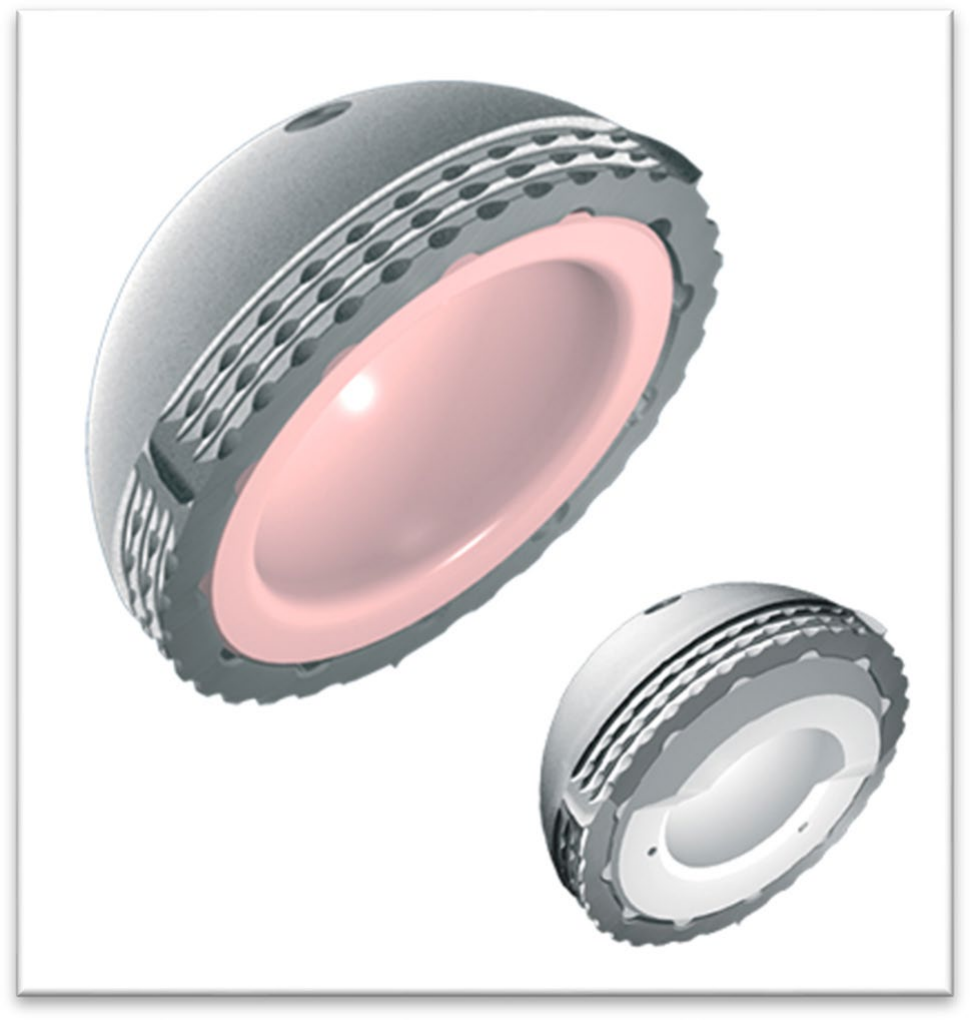
The both types of rigid cups
2. In chromium-cobalt alloy with outside a porous titanium T40 projection, then a layer of 100 μ hydroxyapatite, for the DM variety. (Fig. 5). Its inner surface is polished to obtain an internal Ra of 0.05µ; the inserts for the DM are made of UHMWPE with a maximum roughness of 0.5µ on the outer surface and 1.5µ maximum on the inner surgface; the retention of the clipped head is the one defined according to the usual standards.

**Fig. 5:**
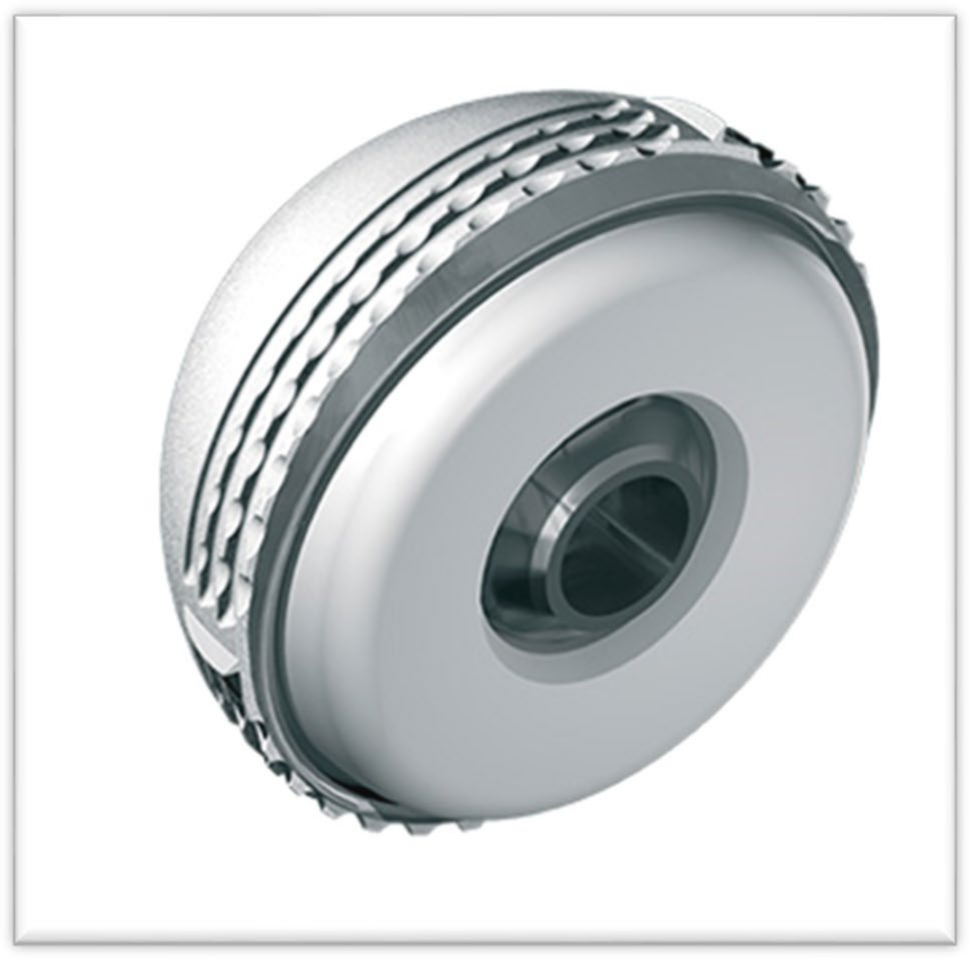
View of a dual-mobility cup

Its inner surface is polished to obtain an internal Ra of 0.05 μ; the inserts for the DM are made of UHMWPE with a maximum roughness of 0.5 μ on the outer surface and 1.5 μ maximum on the inner surface; the retention of this clipped head is the one defined according to the standards.

### Operative technique and choice of implants

A total of 66% were placed posteriorly (1/4 by minimally invasive approach); 34% were placed by anterior approach only when anatomical conditions were favorable to avoid femur difficulties that are significantly correlated with the operative time [21].

The choice of neck angle was based on anatomy: 31 cases (15.3%) with an angle of 122° and 172 cases (84.7%) with an angle of 130°.

73.9% of the heads are ceramic (150) and 26.1% are metallic (56).

Cups were 121 DM (59.6%) and 82 rigid cups (40.4%), with 90.2% of delta ceramic inserts.

The choice of the type of implants (head and cup) depended on physiological age.

Rigid cups have an average age of 56.3 years (26 to 78) with SD 9.4.

MD have an average age of 71.4 years (42 to 90) with SD 9.

The ceramic heads have an average age of 62.3 years with an SD 11.6.

The metal heads (93% steel) have an average age of 73.9 years with an SD 6.6. They were chosen because their results remain good even after 15 to 20 years [22].

### Compliance with ethical standards

#### IRB approval

This is a non-interventional research not involving the human person (RNIPH); the information and non-opposition of patients; and the processing of personal data has been operated in accordance with the Data Protection Act No. 2018–493 of May 3, 2018, and the General Data Protection Regulation, applying the reference methodology MR-004.

#### Conflicts of interest

The authors declare that they received funds because of the licensed patent to FH Ortho, but they received no remuneration for this study.

## Results

### Intraoperative complications

Three fractures (1.5%) of the proximal femur on 130° stems were treated by cerclages and delayed total support for 6 weeks.

### Early complications

Two medically treated hematomas (0.9%) cured without reintervention in four weeks.

One infection (0.4%) required early revision with change of head and cotyloid insert after pressure washing.

One dislocation reduced under general anaesthesia. There was no recurrence at two years, the date of the patient’s lost to follow-up.

### Late complications (3.4%)

**Fig. 6:**
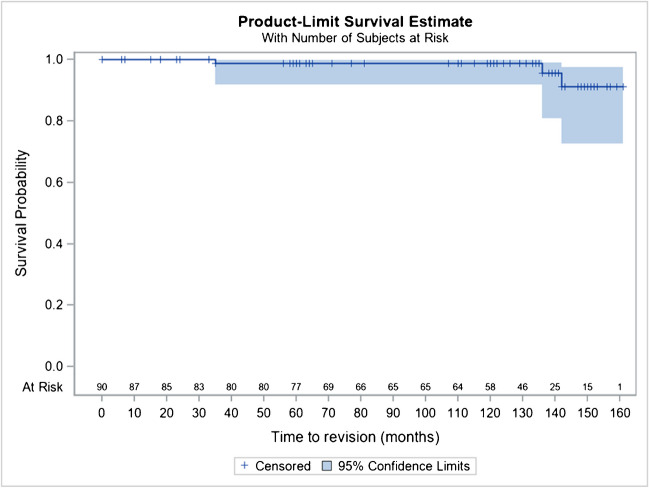
Kaplan–Meier cumulative survival rate, by considering revision for any reason as endpoint, at 10 years is 98.8% (95% CI 91.7–99.8) with a rigid cup

**Fig. 7:**
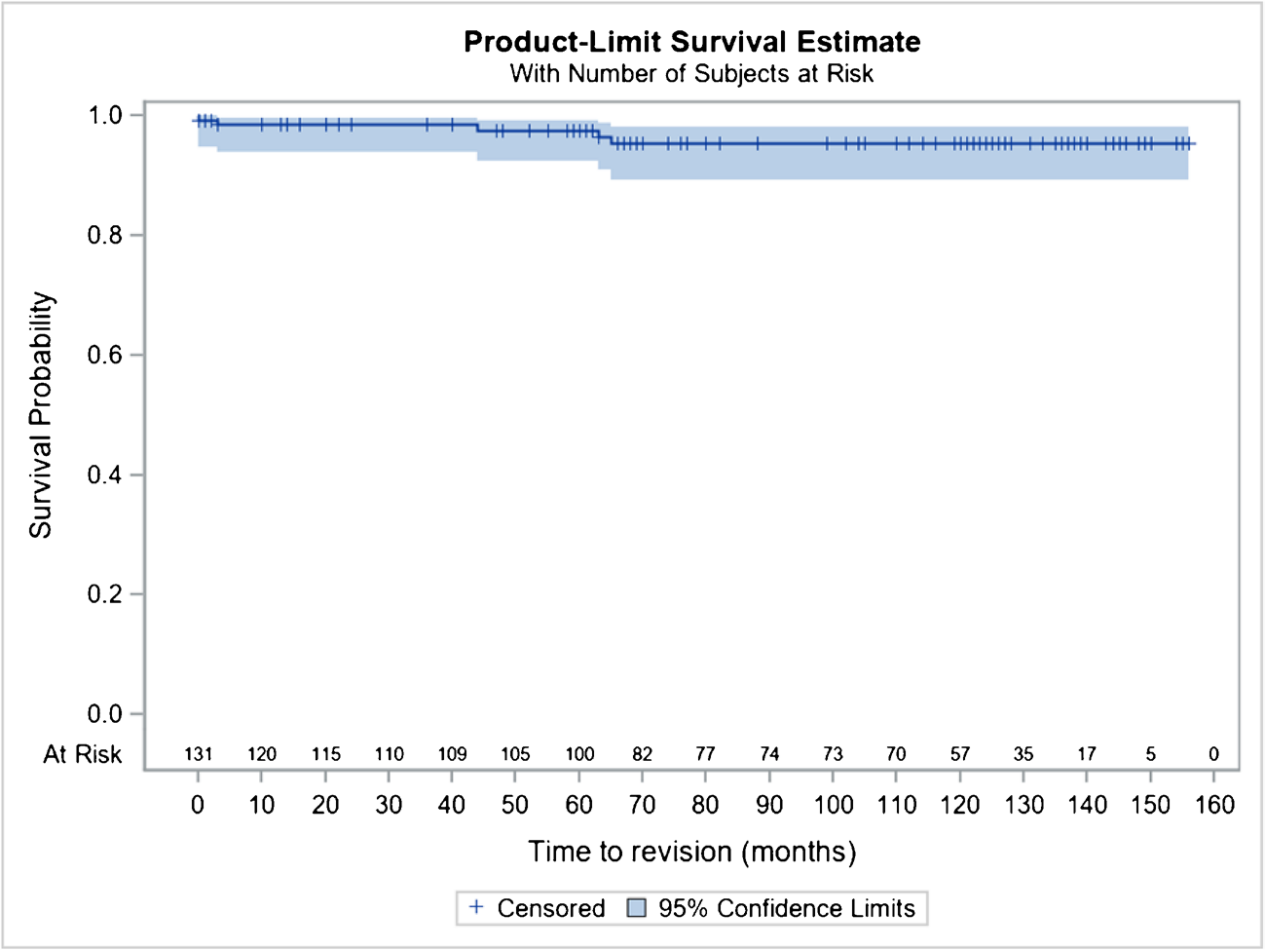
Kaplan–Meier cumulative survival rate, by considering revision for any reason as endpoint, at 10 years is 95.4% (95% CI 89.2–98.1) with a DM cup

Revision of the cup in four cases:

- One traumatic intraprosthetic dislocation at five months: the patient leaning forward, in internal rotating and with his knee extended, lifted a load of 30 kg with sensation of a snap and then pain causing his fall. Revision: change of head and insert since is going well.
- One traumatic intraprosthetic dislocation at six years postoperative, following a jump from a height of 1.50 m. Revision: change of head and insert; PMA at 18/18 to six years.
- One unexplained intraprosthetic dislocation within six years postoperative. There was a surgical revision at another hospital.
- One revision at 11.5 years for cup loosening (after several falls in an 82-year-old woman). There was no other loosening in the series, at ten to 12 years (Figs. 6 and 7).

Revision of the femoral rod in three cases:

- One for periprosthetic fracture on the seventh postoperative day, without real trauma (torsional movement) in a 77-year-old woman; the stem was changed into a recovery cemented stem on cerclage.
- One at 11 years postoperative for persistent femoral mechanical pain on a too small size rod. There was femoral hyperfixation on scintigraphy. Probably, the recently described intraoperative acoustic analysis techniques will prevent this type of complications [23].
- One for post-traumatic periprosthetic fracture after a fall, at ten years postoperative, at age 79.

**Fig. 8:**
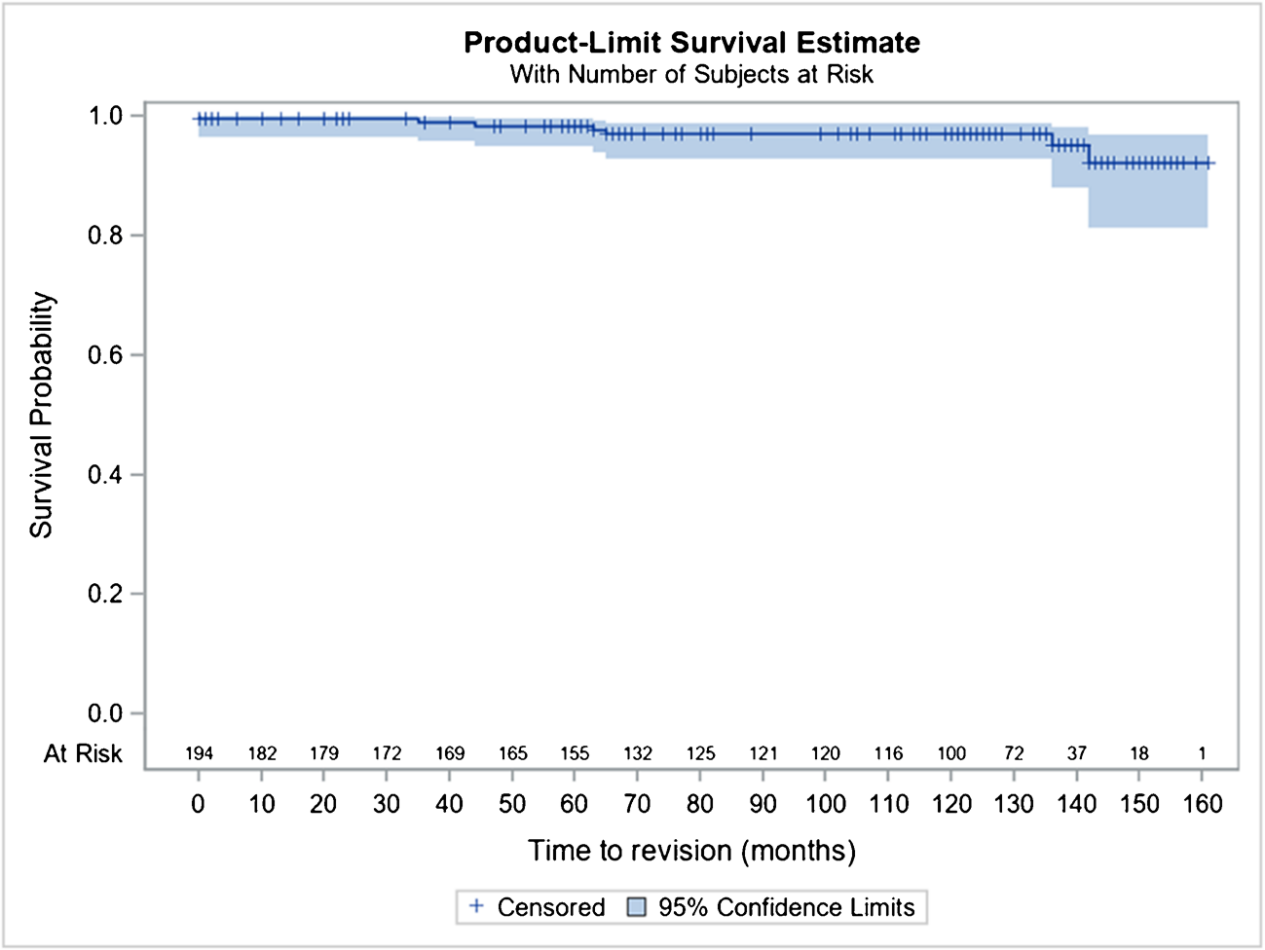
Kaplan–Meier cumulative survival rate of the stem, by considering revision for any reason as endpoint, at 10 years is at 97% (95% CI 92.7–98.7)

**Fig. 9:**
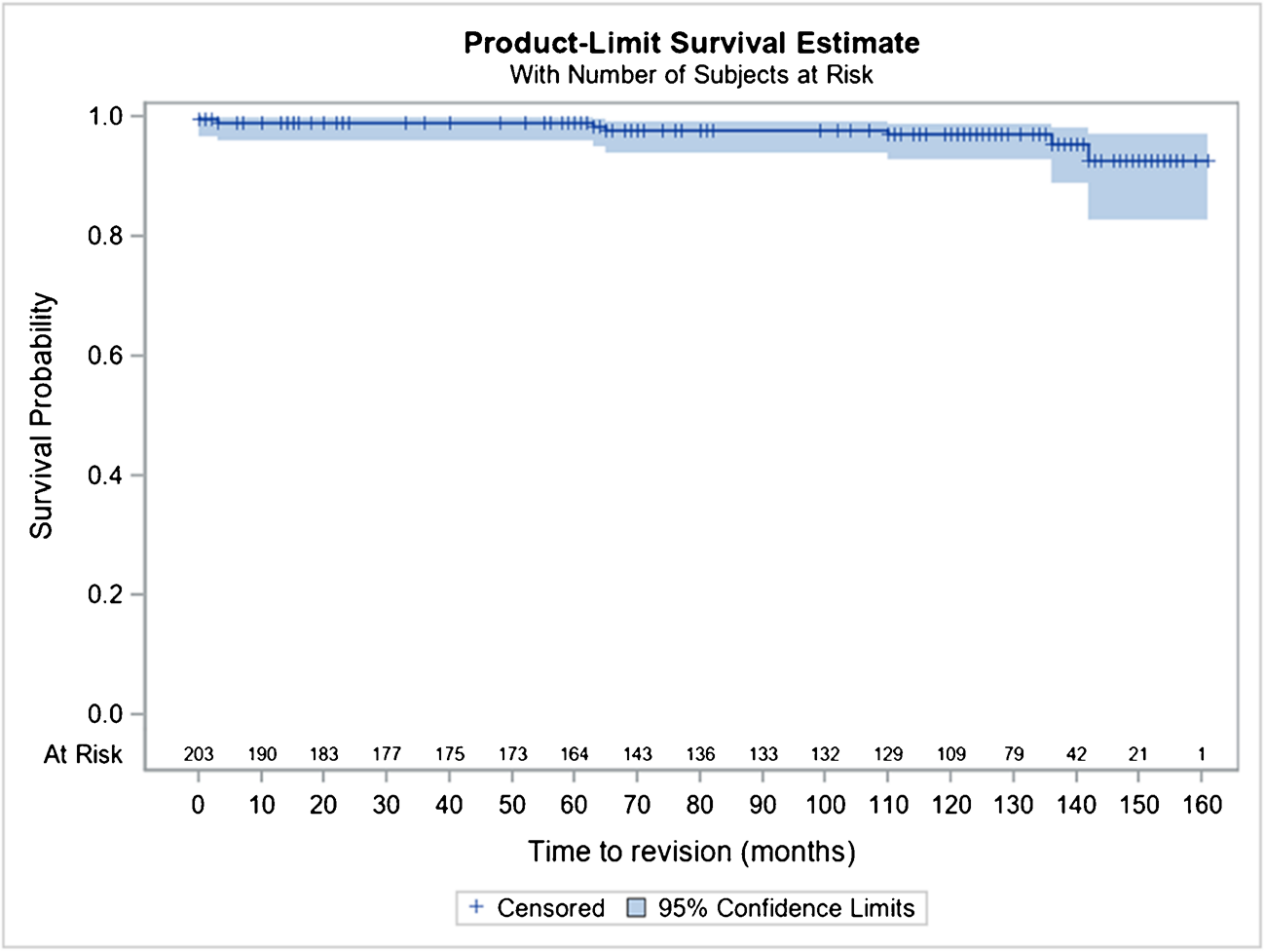
Kaplan–Meier cumulative survival rate of a THR of the series, by considering revision for any reason as endpoint, at 10 years is at 96.6% (95% CI 92.7–98.7)

There is no subsidence or loosening of the stem, nor any other thigh pain reported in the series at ten to 12 years as could be described for cementless rods in femoral neck fracture [24] (Figs. 8 and 9).

## Clinical scores

### Harris score

Evolutions between pre- and postoperative visits are tested with a Wilcoxon rank test. A *p*-value < 0.05 reveals a significant change between the visits.

Harris’ median hip score was preoperative 45.1 (SD 13.5) [95% CI 43.2 to 47.0].

The median 12-year mean hip Harris score is 94.2 (SD 11.9) [95% CI 92.0; 96.4].

There is an average gain of 47.3 (SD 18.8) [95% CI 43.8; 50.8].

The 53 metal heads increase from 54.7 to 84.10, regardless of neck angulation; ceramic heads increase from 45.7 to 96.6, regardless of neck angle.

The choice of the nature of the head is a matter of age: the transition from ceramics to metal is around the age of 70; this age issue probably explains the difference in Harris scores.

The 118 DM cups have Harris score increased from 40.8 to 95.1, with a difference between the 73 ceramic heads (39.4 to 95.8) and the 43 metal heads (43.1 to 90.9).

The 82 rigid cups have Harris score increased from 51 to 93.4.

### PMA score

Evolutions between pre- and postoperative visits are tested with a Wilcoxon rank test. A *p*-value < 0.05 reveals a significant change between the visits.

The median hip PMA score was preoperative 10.3 (SD 2.3) [95% CI 10 to 10.7].

The median 12-year mean hip PMA score is 17.1 (SD 1.9) [95% CI 16.8; 17.5].

There is an average gain of 6.4 (SD 3) [95% CI 5.8; 6.9].

The 53 metal heads (mean age 73.9 years) go from 10.8 to 15.7 in the PMA score; ceramic heads (average age 62.3 years) go from 10.3 to 17.5 in the PMA score.

Age probably has more than the nature of the head to explain the difference in PMA scores between different prosthetic heads.

The 82 rigid cups have their score that goes from 12.4 to 15.1, with a significant difference between polyethylene insert at 15.1 and ceramic insert at 17.5.

The 118 DM cups have their score increase from 9.8 to 17, with a difference between the 73 ceramic heads (9.3 to 17.5) and the 45 metal heads (10.6 to 16).

The choice of the nature of the head in a DM cup seems to have an interest on the functional result at the PMA score, ceramic heads having a slightly better score than metal heads.

## Radiological evaluation

Radiologically, on 86 analyses (68.8% of cases) at ten years and more reported, no significant evolution of the appearance of the cancellous bone around the acetabular cup was noted, nor any ossification.

Forty-seven well-rounded remodelling of the medial cortex of the base of the femoral neck was reported (54.6%).

Rare cortical changes (5.6%) existed at this time of ten years and more:

- Three osteolysis in Gruen zone 6, and one in zone 3 [11].
- Two cortical condensations in zone 3, but without periosteal apposition.
- One Endo medullary cortical thickening in zone 3, and 1 in zone 9. This should probably be avoided if the HA coating is limited to the proximal two-thirds of the stem [25].

Some periprosthetic osteogenesis reactions were noted around the 1/3 distal but no periprosthetic edging.

Finally, there was no reactive line at the metaphyseal level and no osteopenia of the great trochanter.

**Fig. 10:**
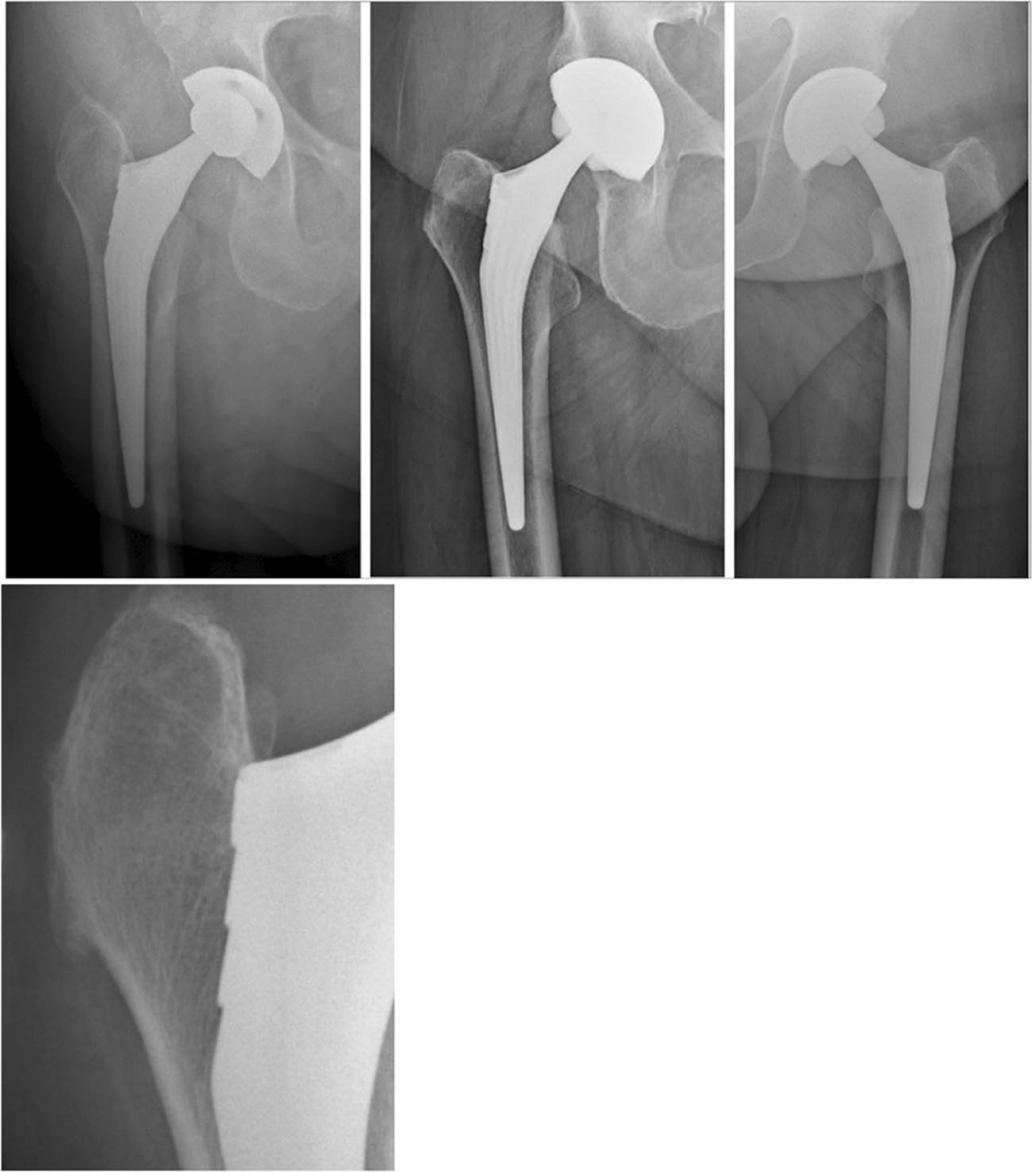
Four examples of an excellent bone integration at ten years follow-up

An uneven length of 3 to 10 mm was measured in 12 cases (Fig. 10).

## Discussion and conclusions

The study is non-comparative and concerns only subjects who have had a cementless stem implanted with a Morse cone of 12/14 and an angulation of 122° or 130°.

The 203 patients included in this study underwent surgery for first-line surgery. The reasons for this primary surgery include only for aetiology primary and secondary coxarthrosis without any cases of osteonecrosis and fracture of the neck of the femur, pathologies where this prosthesis is also regularly used.

A total of 132 patients have a follow-up of at least ten years.

Cementless THR is used more among women, at a rate of 57.8%.

The mean age at surgery is 65.3 years (SD = 11.7; range 26–90).

The mean BMI was 27 kg/m^2^ (SD = 4.7; range 17–48).

The performance assessment is mainly based on the Harris and PMA scores between the preoperative period and the postoperative period performed with a follow-up of at least ten years.

The total Harris score improved significantly (*p*-value < 0.0001) from a mean preoperative score of 45.1 (SD = 13.5; range 43.2–47) to a mean postoperative score, over ten years or more, at 94.2 (SD = 11.9; range 92–96.4).

The clinically important minimum difference (MCID) is the smallest change in a treatment outcome that a patient would identify as such [19, 26]. This clinically felt minimal difference (MCID) is an important concept used to determine whether an intervention improves perceived patient outcomes. The National Institute for Health and Care Excellence (NICE) guidelines provide information regarding this MCID. The MCID values reported for the Harris hip score are as follows: 7–10.

In our study, this MCID for the Harris score was obtained for 96.7% of the population at control of ten years or more compared to the preoperative state.

For the population with ten years or more between the PMA score improved significantly (*p*-value < 0.0001) from a mean preoperative score of 10.7 (SD = 1.9; range 95%: 10.3–11.1) to a mean postoperative score at ten years or later at 17.1 (SD = 1.9; range 95%: 16.8–17.5). All subscores of the PMA score (i.e., pain, walking-walking activities, and mobility) show a significant improvement (*p* < 0.0001) between preoperative and postoperative status.

The safety of using these implants was evaluated by measuring the complication rate, revision rate, and survival analysis, according to Kaplan–Meier.

The rate of intraoperative complications is 1.5%.

The rate of postoperative complications requiring revision was calculated at 3.4% with a 95% confidence interval at least ten years. These are below the current threshold recommended by NICE which is 5% maximum at ten years.

In addition, considering revision for any reason as an endpoint, Kaplan–Meier’s analyses estimated a cumulative survival rate of the prosthesis at ten years (120 months) at 97% (95% CI 92.7–98.7%), considering the Hip and Go prosthesis as a whole.

In comparison, cumulative revision rates for primary hip replacement (all types of THR) were extracted from the Australian Registry 2022 (between 4.9 and 5.1%) [Australian Orthopedic Association National Joint Replacement Registry Annual Report 2022], the English Registry 2020 (3.7%) [England Registry 2020/Table 3.H5 page 58–min and max of lines “All cemented,” “All uncemented,” “All hybrid,” and “All reverse hybrid”], and the Swedish Registry 2019 (4.3%) [Swedish Hip Arthroplasty Register Annual report 2019].

Given our cumulative ten-year revision rate of 3.4%, it is therefore fair to say that the prosthesis used in our series is associated with a revision rate at least comparable, even a little lower, than some data from the world literature of high scientific level as exhaustive registers of several thousand cases [Australia, England, and Swedish mentioned above].

The NICE stated that THRs are recommended as treatment options for people with coxarthrosis if, and only if, the prostheses have revision rates of 5% or less at ten years. It is therefore possible to conclude that THR with Hip and Go implantable medical devices is recommended as a treatment for osteoarthritis.

Thus, considering the safety results and functional scores presented above, it seems possible to conclude that these devices used in the placement of a THR in our series are safe devices allowing functional improvement between pre- and postoperative states.

In addition, subgroup analyses by rod CC’D angle, torque version, or head type are also provided in this report for their performance (Harris and PMA scores) and for their safety (survival rate, revision rate, complications) with some differences related more to the field of age, than to the implants themselves.

- HNG SC stem 122°: 92.0% (95% CI 70.8–98.0%)
- HNG SC stem 130°: 97.0% (95% CI 92.8–98.7%)
- HNG rigid cut: 98.8% (95% CI 91.7–99.8%)
- HNG DM SC press-fit cup: 95.4% (95% CI 89.2–98.1%)

However, this study has some limitations. These limitations are those of any study, retrospective or prospective: they are mainly missing data, especially the high rate of patients lost to follow-up at ten years (21.7% *n* = 44/203) and the rate of patients identified as deceased (16.7% *n* = 34/203).

Finally, these results are limited by the number of patients seen at follow-up visits (125/203 patients with follow-up for at least ten years, and all having had an intermediate visit at five years). This deficit in the number of follow-ups can have an impact on the calculation of survival rate and confidence intervals. In addition, due to the absence of a control group and the absence of double blinding, the following methodological biases, classic and inherent in any study, must be taken into account: confounding bias (related to failure to account for confounders), monitoring, and evaluation bias (related to lack of blinding and choice of outcomes potentially affected by patient autosuggestion and assessor subjectivity).

Due to the various constraints related to the evaluation of an implanted device, these biases are unfortunately still present in published studies in medical field. However, despite the limitations mentioned above, our study can be considered to allow an evaluation of the device (Hip and Go THR) that is representative of current practice.

## Data Availability

There is a total availability of data and material (data transparency).
